# Supplementary material for: Predictors of Psychological Distress among Post-Operative Cardiac Patients: A Narrative Review
Source: Healthcare (Basel). 2023 Oct 12;11(20):2721. doi: 10.3390/healthcare11202721 (PMC10606887; doi:10.3390/healthcare11202721)
Supplement: Supplementary file 1 [file healthcare-11-02721-s001.zip › healthcare-2633795-supplementary.pdf]

Supplementary Table S1

Summary of Reviewed Studies by Related Factors

Author (year) and country	Search Methodology used	Methodological design	Intervention content	Control content	Interesting findings
		Personal factors			
Perski et al. (1998) / Sweden	Database search	Prospective observational study<br>• T0: baseline pre-operation<br>• T1: one-year post-operation<br>• T2: three years post-operation	-	-	Patients with elevated psychological distress pre-operation has higher symptoms at T1 and higher rate of cardiac events at T2
Heijmeriks et al. (1999) / The Netherlands	Database search	Prospective observational study<br>• T0: baseline (pre-operation)<br>• T1: two months post-operation<br>• T2: six months post-operation	-	-	Elderly and younger patients have same surgical and one-year mortality rate<br>Both had improved quality of life responses
Peterson et al. (2002) / United States of America	Database search	Prospective observational study<br>• T0: baseline pre-operation<br>• T1: six months post-operation<br>• T2: 36 months post-operation	-	-	Increase in depression at six months post-operation was associated with cardiac events in the following period
Jackson et al. (2005) / Canada	Citation search	Quantitative review	-	-	Patients most likely to attend cardiac rehabilitation with physician endorsement, patient has a high self-efficacy, easily accessible, patient is educated and is married<br>Patients least likely to attend

Author (year) and country	Search Methodology used	Methodological design	Intervention content	Control content	Interesting findings
					when rehabilitation requires long distance travel
Gravely-Witte et al. (2007) / Canada	Database search	Prospective observational study<br>• T0: baseline (45 days post-hospital admission)<br>• T1: six months post-hospital admission	-	-	Higher self-reported levels of depression were associated with past cardiac history at six months post-operation<br><br>Angina was associated with higher self-reported levels of depression, worse emotional, physical and social health-related quality of life scores
Gjeilo et al. (2008) / Norway	Database search	Prospective observational study<br>• T0: baseline<br>• T1: six months post-operation<br>• T2: one-year post-operation	-	-	Female patients reported lower quality of life scores than male patients (pre- and post-operation)<br>Overall improvement in quality-of-life scores post-surgery<br>Difference in sex most evident at T1
Whooley et al. (2008) / United States of America	Database search	Prospective observational study<br>• T0: baseline<br>• T1: one-year post-recruitment<br>• T2+: Annual check-ups until study ceased eight	-	-	Depression was associated with future cardiac events<br>Physical inactivity mediates the association between depression and cardiac events

Author (year) and country	Search Methodology used	Methodological design	Intervention content	Control content	Interesting findings
		years after commencement			
McKenzie et al. (2010) / England	Database search	Systematic review	-	-	Depressive and anxiety symptoms best predicted by pre-operative measures<br>Gender and age were inconclusive predictors
Morone et al. (2010) / United States of America	Database search	RCT<br>• T0: baseline (two weeks post-hospitalisation)<br>• T1: two months post-operation<br>• T2: 12 months post-operation	Depressed intervention consisted of eight months of:<br>(a) telephone calls to patients to arrange care (e.g., workbooks on depression, medication, reference to mental health specialist)<br>(b) weekly case reviews with nurse care manager, study psychiatrist and internist	Usual care	Depressed patients had worse pain scores compared to non-depressed<br>Collaborative care associated with better pain scores among depressed patients
Kendel et al. (2011) / Germany	Database search	Prospective observational study<br>• T0: baseline pre-operation<br>• T1: one-year post-operation	-	-	Both men and women reported improved quality of life scores<br>Gender was an independent marker for role functioning
Herring et al. (2012) / United States of America	Database search	Systematic review and meta-analysis	-	-	Exercise reduced depression among chronic patients<br>This was most apparent among

Author (year) and country	Search Methodology used	Methodological design	Intervention content	Control content	Interesting findings
					patients with high baseline depression scores
Horne et al. (2013a) / Canada	Citation search	Prospective observational study<br>• T0: baseline (pre-operation)<br>• T1: hospital discharge<br>• T2: three months post-operation<br>• T3: six months post-operation	-	-	Depression was independently associated with physical inactivity, pre-operative depression, over seven days in hospital post-operation, stressful post-operative events, pre-operative left ventricular ejection fraction < 50% and cardiopulmonary bypass time > 120 minutes<br>Physical inactivity associated with both pre-operative depression and post-operative depression at six months
Horne et al. (2013b) / Canada	Database search	Prospective observational study<br>• T0: baseline (pre-operation)<br>• T1: hospital discharge<br>• T2: three months post-operation<br>• T3: six months post-operation	-	-	Depression was independently associated with physical inactivity, pre-operative depression, over seven days in hospital post-operation, stressful post-operative events, pre-

Author (year) and country	Search Methodology used	Methodological design	Intervention content	Control content	Interesting findings
					operative left ventricular ejection fraction < 50% and cardiopulmonary bypass time > 120 minutes<br>Physical inactivity associated with both pre-operative depression and post-operative depression at six months
Tamis-Holland et al. (2013) / United States of America	Database search	Randomised 2x2 factorial study<br>• T0: baseline<br>• T1: one-year post-discharge<br>• T2: two-year post-discharge<br>• T3: three-year post-discharge<br>• T4: four-year post-discharge<br>• T5: five-year post-discharge	Prompt revascularization plus intensive medical therapy *versus* Initial intensive medical therapy alone with clinically indicated revascularization<br>Insulin-providing *versus* Insulin-sensitizing strategy	-	Women reported more angina than men
Fiuza-Luces (2018) / Spain	Database search	Narrative literature review	-	-	Exercise healthy level of anti-inflammatory milieu, myocardial regeneration, and ameliorates age-related loss of muscle mass and strength

Author (year) and country	Search Methodology used	Methodological design	Intervention content	Control content	Interesting findings
Sanyal et al. (2018) / India	Database search	Between-groups observational study<br>• T1: three months post-operation	-	-	Resilience (recovery to normal functioning) is a predictor of increased quality of life scores in 'older' patients (55 to 70 years)<br>'Older' patients had greater resilience scores than 'younger' patients (30 to 45 years)
Zaini et al. (2022) / Malaysia	Database search	Cross-sectional study<br>• T1: pre-operation	-	-	Older participants associated with greater proportion of psychological distress<br>Males had higher proportion of depression compared to females
Social factors					
Jenkins et al. (1983) / United States of America	Database search	Prospective observational study<br>• T0: baseline pre-operation<br>• T1: six months post-operation	-	-	Psychological distress, wellbeing, fatigue and sleep improved from pre-operative levels
Gilliss (1984) / United States of America	Database search	Prospective observational study<br>• T0: baseline (week post-hospitalisation)	-	-	Patient spouses has higher self-reported levels of stress compared to patients at hospitalisation<br>At six months, patients and

Author (year) and country	Search Methodology used	Methodological design	Intervention content	Control content	Interesting findings
		• T1: six months post-operation			spouses reported high levels of marital conflict
Artinian (1993) / United States of America	Database search	Prospective exploratory study<br>• T1: six weeks post-surgery	-	-	Perceived readiness for hospital discharge was predicted by patients' personal resources, social support, knowledge, and utilisation of coping strategies<br>The key influencers on patient partners were physicians, nurses, immediate family, and friends with experience of cardiac surgery
Moser et al. (1993) / United States of America	Citation search	Prospective observational study<br>• T1: five months post-hospitalisation	-	-	Patients and their partners stated that information was the most important need<br>Patients and partners differed on the importance of other needs<br>Most needs (40% to 70%) were not met by nurses or physicians
Oxman et al. (1995) / United States of America	Database search	Prospective observational study<br>• T0: baseline pre-operation	-	-	Lack of participation in social groups and absence of strength and comfort in

Author (year) and country	Search Methodology used	Methodological design	Intervention content	Control content	Interesting findings
		• T1: one-month post-operation<br>• T2: six months post-operation			religion are two predictors of mortality
Goodman (1997) / England	Database search	Retrospective interview study<br>• Interview at six-weeks post-operation<br>• Diary entries during six weeks post-operation	-	-	There were many issues for patients and not many were the same which suggests tailored approach is most suitable<br>Patients expressed desire for social interaction<br>Mixed psychological states between patients<br>Patients were mostly all informed of exercise knowledge
Davies (2000) / England	Database search	Prospective observational study<br>• T1: one-week post-discharge<br>• T2: seven weeks post-discharge	-	-	Higher satisfaction with information scores were associated with lower levels of anxiety and depression
Robles & Kiecolt-Glaser (2003) / United States of America	Database search	Narrative review	-	-	Marital functioning is associated with physiological changes which in turn can influence long-term cardiovascular health
Eng et al. (2011) / Malaysia	Database search	Systematic review and meta-analysis	-	-	Unmarried participants with

Author (year) and country	Search Methodology used	Methodological design	Intervention content	Control content	Interesting findings
					cardiovascular disease were associated with higher self-reported depression compared to married participants<br>Comorbidity with other diseases was associated with higher self-reported depression
De Fazio et al. (2012) / Italy	Database search	Cross-sectional study<br>• T1: within one-week post-surgery	-	-	Self-reported depression was associated with b-blocker therapy, Type D personality, and specific coping strategies<br>Self-reported anxiety was independently predicted by unmarried status, low education, unstable angina, Type D personality, emotion, and avoidance-oriented coping
Bucholz et al. (2014) / United States of America	Database search	Prospective longitudinal study<br>• T0: baseline<br>• T1: one-month post-hospitalisation<br>• T2: 12 months post-hospitalisation	-	-	Lower perceived social support was associated with increased depression at 12 months post-operation

Author (year) and country	Search Methodology used	Methodological design	Intervention content	Control content	Interesting findings
Manemann et al. (2018) / United States of America	Database search	Prospective observational study<br>• T0: baseline<br>• T1: one month after initial survey<br>• T2: two months after initial survey	-	-	Patients with high social isolation had 3.5 times greater risk of death<br>Patients with moderate social isolation were not associated with increased risk of death or hospitalisation
Nielsen et al. (2019) / Sweden	Database search	Cross-sequential study<br><br>Used the Swedish Web-system for Enhancement and Development of Evidence-Based Care in Heart Disease Evaluated According to Recommended Therapies for recruitment	-	-	Patients who were never married had a higher risk of mortality if they were women compared to men<br>Higher risk of mortality in lower income men than women when comparing to higher income patients
Caccamo et al. (2020) / Italy	Database search	Prospective observational study<br>• T0: baseline before cardiac rehabilitation<br>• T1: two weeks later (post-rehabilitation)	-	-	Marital status was associated with different self-reported psychological distress outcomes post-operation<br>Unmarried participants had greater social inhibition and anxiety scores compared to married or cohabitating and divorced

Author (year) and country	Search Methodology used	Methodological design	Intervention content	Control content	Interesting findings
					or widowed participants
Dhindsa et al. (2020) / United States of America	Database search	Narrative literature review	-	-	Unmarried patients with cardiovascular disease have an increased rate of further cardiac events compared to married participants
		Health service factors			
Asilioglu & Celik (2004) / Turkey	Database search	Prospective experimental study<br>• T1: three days post-surgery	Effect of pre-operative education on post-operative anxiety	Usual care informed of pre- and post-operative routines	No statistical differences in either state or trait anxiety compared to control
Linden et al. (2007) / Canada	Database search	Meta-analysis	-	-	Psychological treatment was associated with reduced mortality and cardiac events<br>Mortality benefits was found only in males
Shelley & Pakenham (2007) / Australia	Database search	Single-blind randomised study<br>• T0: baseline (pre-operation)<br>• T1: day of hospital discharge	-	-	High self-efficacy locus of control, preparation was related to lower distress for high self-efficacy participants compared to low self-efficacy<br>Low self-efficacy locus of control, preparation was related to lower distress for low self-efficacy participants

Author (year) and country	Search Methodology used	Methodological design	Intervention content	Control content	Interesting findings
					compared to lower self-efficacy
Sørlie et al. (2007) / Norway	Citation search	RCT<br>• T0: baseline<br>• T1: discharge from hospital<br>• T2: two weeks post-discharge<br>• T3: six weeks post-discharge<br>• T4: six months post-discharge<br>• T5: one-year post-discharge<br>• T6: two years post-discharge	Video information, individualised information by nurses. Carried out at hospital admission and discharge.	Standardised information with no video.	Patients from the intervention group had less anxiety at discharge to a year post-discharge compared to control<br>Intervention patients reported less depression from six months to two years post-discharge
Sharif et al. (2012) / Iran	Database search	RCT<br>• T0: baseline (discharge from hospital)<br>• T1: immediately post-rehabilitation<br>• T2: two months post-rehabilitation	Eight cardiac rehabilitation sessions over four weeks	Given usual care and education booklet	Depression scores were reduced for patients in the intervention group<br>Anxiety scores were not different between control and intervention groups
Okamoto & Motomura (2017) / Japan	Database search	Narrative review	-	-	Limited number of studies investigating depression and anxiety among patients beyond six months post-operation<br>Patients improved psychological distress scores between three to six months post-operation

Author (year) and country	Search Methodology used	Methodological design	Intervention content	Control content	Interesting findings
Ng et al. (2021) / Singapore	Database search	Systematic review and meta-analysis	-	-	Pre-operative education associated with reducing pre-operation anxiety, length of stay in intensive care units and improving knowledge<br>Lower depression, post-operative anxiety and higher satisfaction were also associated with pre-operative education but to a smaller effect

Supplementary Table S2

Search Terms for Database Searches

Date	Database	Search strategy	Result number	Field search	Limits / Filters	Notes
16/02/2022	Ebscohost Megafile Ultimate. Includes: Academic Search Ultimate, APA PsycArticles, APA PsycInfo, CINAHL with Full Text, E-Journals, Psychology and Behavioral Sciences	(cardiac patient?) AND (depression OR “mental health” OR “mental illness” OR anxiet* OR anxious OR stress* OR distress*) AND (“post-operative” OR postoperative OR postop OR “post surgery” OR “after surgery” OR “following surgery”)	812	Title-abstract -subject	English language	Example of a proximity search; note that there might be duplicates due to searching multiple databases. Results indicated the keyword stress was relating more to physiological stress factors - try removing the term.
16/02/2022	Ebscohost Megafile Ultimate.	(cardiac N5 patient?) AND (depression OR “mental health” OR “mental illness” OR anxiet* OR anxious OR distress*) AND (“post-operative” OR postoperative OR postop OR “post surgery” OR “after surgery” OR	525	Title-abstract -subject	English language	Removed stress as keyword for concept #2.

Date	Database	Search strategy	Result number	Field search	Limits / Filters	Notes
		“following surgery”)				
16/02/2022	Ebscohost Megafile Ultimate.	(“cardiac patient” OR “cardiac patients") AND (depression OR “mental health” OR “mental illness” OR anxiet* OR anxious OR stress* OR distress*) AND (“post-operative” OR postoperative OR postop OR “post surgery” OR “after surgery” OR “following surgery”)	355	All	None	There might be duplicates due to searching multiple databases.
16/02/2022	Ebscohost Megafile Ultimate.	(“cardiac patient” OR “cardiac patients") AND (depression OR “mental health” OR “mental illness” OR anxiet* OR anxious OR stress* OR distress*) AND (“post-operative” OR postoperative OR postop OR “post surgery” OR	315	All	English language	N/A

Date	Database	Search strategy	Result number	Field search	Limits / Filters	Notes
		"after surgery" OR "following surgery")				
21/03/2022	Ebscohost Megafile Ultimate.	("cardiac patient" OR "cardiac patients") AND (depression OR "mental health" OR "mental illness" OR anxiet* OR anxious OR stress* OR distress*) AND ("post-operative" OR postoperative OR postop OR "post surgery" OR "after surgery" OR "following surgery")	104	Title-abstract-subject	English language	N/A
21/02/2022	Cochrane Trials. Includes: PubMed, Embase, CT.gov, ICTRP, CINAHL	(cardiac patient OR cardiac patients) AND (depression OR mental health OR mental illness OR anxiet* OR anxious OR stress* OR distress*) AND (post-operative OR postoperative OR postop	1946	Title-abstract-subject	None	N/A

Date	Database	Search strategy	Result number	Field search	Limits / Filters	Notes
		OR post surgery OR after surgery OR following surgery)				
21/02/2022	Cochrane Reviews	(“cardiac patient” OR “cardiac patients") AND (depression OR “mental health” OR “mental illness” OR anxiet* OR anxious OR stress* OR distress*) AND (“post-operative” OR postoperative OR postop OR “post surgery” OR “after surgery” OR “following surgery”)	0	Title-abstract-subject	None	Was not able to successfully search due to the quotation marks.
21/02/2022	Cochrane Reviews	(cardiac patient OR cardiac patients) AND (depression OR mental health OR mental illness OR anxiet* OR anxious OR stress* OR distress*) AND (post-operative OR	27	Title-abstract-subject	None	N/A

Date	Database	Search strategy	Result number	Field search	Limits / Filters	Notes
		postoperative OR postop OR post surgery OR after surgery OR following surgery)				
21/02/2022	Cochrane Reviews	(cardiac patient OR cardiac patients) AND (depression OR mental health OR mental illness OR anxiet* OR anxious OR stress* OR distress*) AND (post-operative OR postoperative OR postop OR post surgery OR after surgery OR following surgery)	28	Title-abstract-subject	None	N/A
23/02/2022	Springer	(“cardiac patient” OR “cardiac patients") AND (depression OR “mental health” OR “mental illness” OR anxiet* OR anxious OR stress* OR distress*) AND (“post-	0	All	None	Was not able to successfully search using these terms.

Date	Database	Search strategy	Result number	Field search	Limits / Filters	Notes
		operative" OR postoperative OR postop OR "post surgery" OR "after surgery" OR "following surgery")				
23/02/2022	Springer	(cardiac patient OR cardiac patients) AND (depression OR mental health OR mental illness OR anxiet* OR anxious OR stress* OR distress*) AND (post-operative OR postoperative OR postop OR post surgery OR after surgery OR following surgery)	130	All	None	N/A
23/02/2022	Springer	(cardiac patient OR cardiac patients) AND (depression OR mental health OR mental illness OR anxiet* OR anxious OR stress* OR	109	All	Article, chapter and conference paper	N/A

Date	Database	Search strategy	Result number	Field search	Limits / Filters	Notes
		distress*) AND (post-operative OR postoperative OR postop OR post surgery OR after surgery OR following surgery)				
23/02/2022	ScienceDirect	(“cardiac patient” OR “cardiac patients") AND (depression OR “mental health” OR “mental illness” OR anxiet* OR anxious OR stress* OR distress*) AND (“post-operative” OR postoperative OR postop OR “post surgery” OR “after surgery” OR “following surgery”)	0	All	None	Was not able to successfully search. Error message: Max. of 8 boolean connectors per field.
23/02/2022	ScienceDirect	(“cardiac patient”) AND (depression OR anxiet* OR anxious OR stress* OR distress*) AND (“post-operative”	0	All	None	Was not able to successfully search. Error message: Wildcards '*' not supported.

Date	Database	Search strategy	Result number	Field search	Limits / Filters	Notes
		OR “post surgery” OR “following surgery”)				
23/02/2022	ScienceDirect	(“cardiac patient") AND (depression OR anxiety OR anxious OR stress OR distress) AND (“post-operative” OR “post surgery” OR “following surgery”)	3740	All	None	N/A
4/03/2022	ScienceDirect	(“cardiac patient") AND (depression OR anxiety OR anxious OR distress) AND (“post-operative” OR “post surgery” OR “following surgery”)	2752	All	None	N/A
4/03/2022	ScienceDirect	(“cardiac patient") AND (depression OR anxiety OR anxious OR distress) AND (“post-operative” OR “post surgery” OR “following surgery”)	1953	All	Review articles, research articles, conference abstracts	N/A
4/03/2022	ScienceDirect	(“cardiac patient") AND (depression	19	Title-abstract -	None	N/A

Date	Database	Search strategy	Result number	Field search	Limits / Filters	Notes
		OR anxiety OR anxious OR stress OR distress) AND ("post-operative" OR "post surgery" OR "following surgery")		keywords		
22/02/2022	SAGE Journals	("cardiac patient" OR "cardiac patients") AND (depression OR "mental health" OR "mental illness" OR anxiet* OR anxious OR stress* OR distress*) AND ("post-operative" OR postoperative OR postop OR "post surgery" OR "after surgery" OR "following surgery")	0	All	None	Unable to successfully search. Error message: Service inaccessible The IP address 139.86.7.217 has been blocked.
